# Supplementary figures and images for: Cryptococcus neoformans adapts to host CO2 concentrations via metabolic and stress-response remodeling
Source: PLoS Biol. 2026 May 5;24(5):e3003561. doi: 10.1371/journal.pbio.3003561 (PMC13160432; doi:10.1371/journal.pbio.3003561)

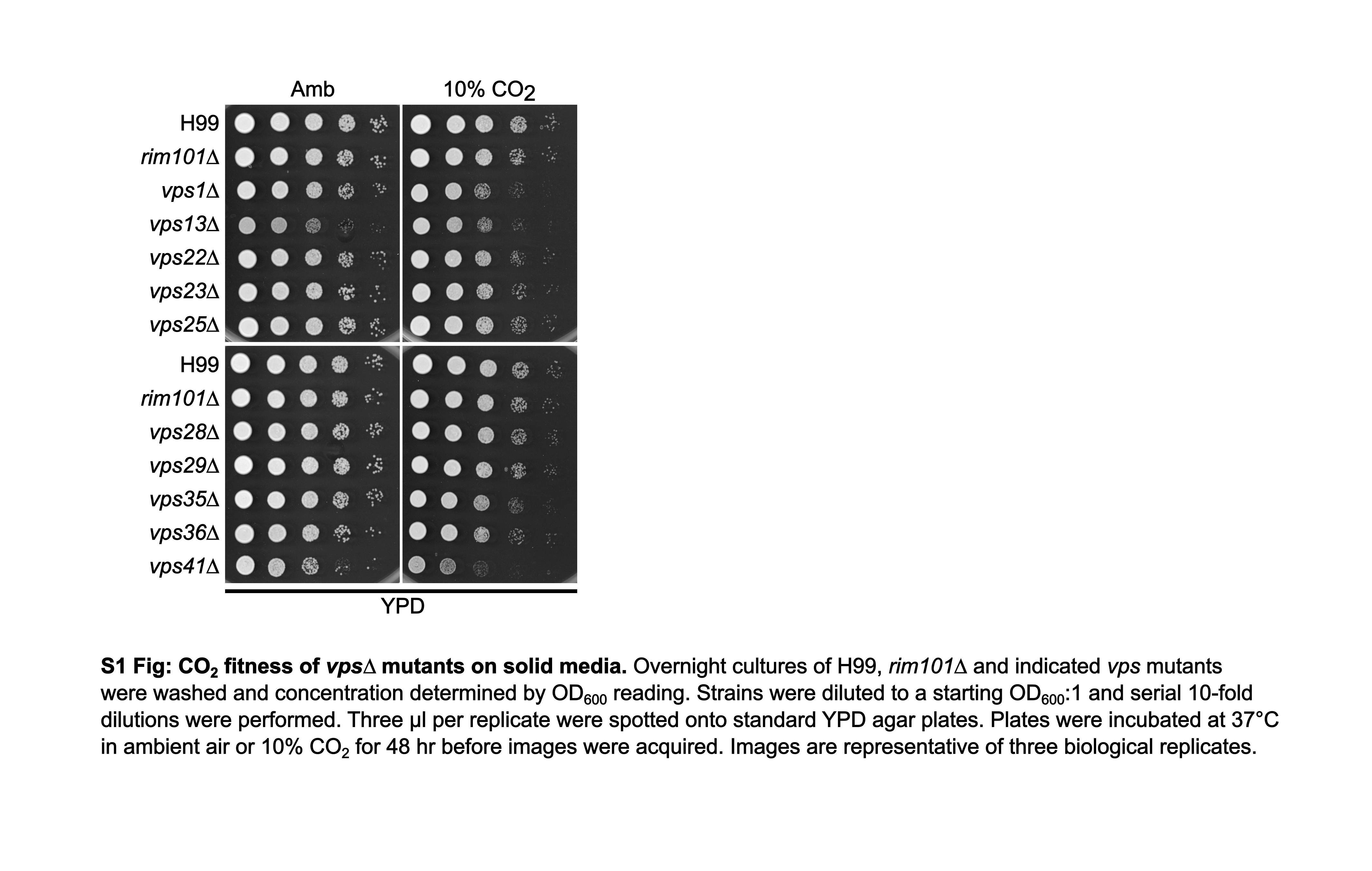

Supplement: S1 Fig — (TIFF) [file pbio.3003561.s001.tiff]
