# Supplementary material for: Cryptococcus neoformans adapts to host CO2 concentrations via metabolic and stress-response remodeling
Source: PLoS Biol. 2026 May 5;24(5):e3003561. doi: 10.1371/journal.pbio.3003561 (PMC13160432; doi:10.1371/journal.pbio.3003561)
Supplement: S1 Raw Images — (PDF) [file pbio.3003561.s007.pdf]

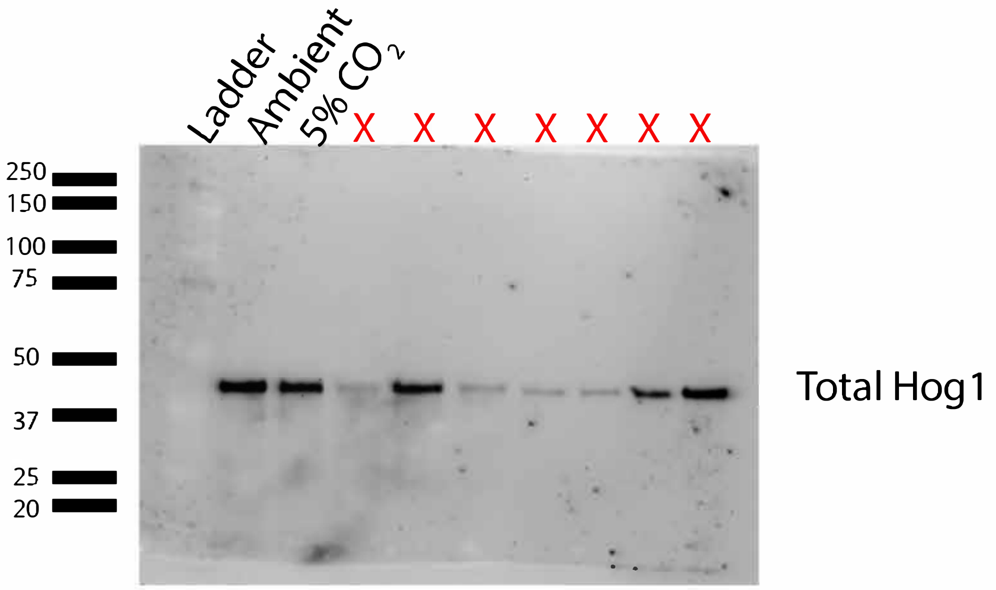

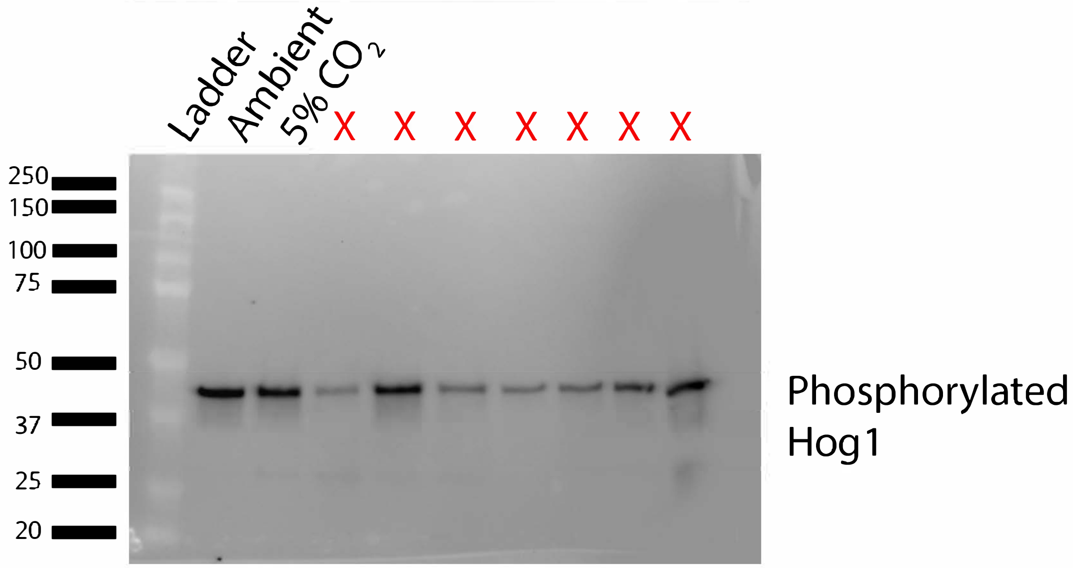

Fig 1C. Images were acquired on a Thermo myECL imager . Total Hog1 was acquired with a 30 minute exposure. Phosphorylated Hog1 was acquired with a 1 minute exposure.
